# Silver(I) Complexes with Thiosalicylic Acid Derivatives as Promising Antimicrobial, Antibiofilm, and Immunomodulatory Agents

**DOI:** 10.3390/pharmaceutics18101213

**Published:** 2026-09-24

**Authors:** Jovana Marinković, Milena Jurišević, Nevena Gajović, Ivan Jovanović, Jovana Z. Marinković, Mirjana Ž. Grujović, Katarina G. Marković, Ivana D. Radojević, Marina Vesović, Miloš Nikolić, Dušan Tomović, Gordana Radić, Nikola Nedeljković

**Affiliations:** 1 Faculty of Medicine of the Military Medical Academy, University of Defence, Crnotravska 17, 11000 Beograd, Serbia; jovana.eko@gmail.com; 2 Center for Molecular Medicine and Stem Cell Research, Faculty of Medical Sciences, University of Kragujevac, Svetozara Markovića 69, 34000 Kragujevac, Serbia; gajovicnevena@yahoo.com (N.G.); ivanjovanovic77@gmail.com (I.J.); jovanamarinkovic266@gmail.com (J.Z.M.); 3 Department of Pharmacy, Faculty of Medical Sciences, University of Kragujevac, Svetozara Markovića 69, 34000 Kragujevac, Serbia; milos.nikolic@fmn.kg.ac.rs (M.N.); dusantomovic88@hotmail.com (D.T.); vasic_gordana@yahoo.com (G.R.); nikola.nedeljkovic@fmn.kg.ac.rs (N.N.); 4 Faculty of Medicine, University of East Sarajevo, Studentska 5, 73300 Foca, Bosnia and Herzegovina; 5 Institute of Public Health Kragujevac, 34000 Kragujevac, Serbia; 6 Department of Science, Institute for Information Technologies, University of Kragujevac, Liceja Kneževine Srbije 1a, 34000 Kragujevac, Serbia; mirjana.grujovic@pmf.kg.ac.rs (M.Ž.G.); katarina.mladenovic@pmf.kg.ac.rs (K.G.M.); 7 Department of Biology and Ecology, Faculty of Science, University of Kragujevac, Radoja Domanovića 12, 34000 Kragujevac, Serbia; ivana.radojevic@pmf.kg.ac.rs

**Keywords:** silver(I) complexes, thiosalicylic acid, antimicrobial, immunomodulatory effect

## Abstract

**Background/Objectives:** Silver has long been used in medicine for its diverse effects. Coordination of this metal ion with bioactive ligands such as thiosalicylic acid derivatives allows the synthesis of potentially promising antimicrobial and antitumor agents. **Methods:** The in vitro antimicrobial activity of silver(I) complexes (**C1**–**C5**) and their corresponding ligands (**L1**–**L5**) was determined using a resazurin-based microdilution method, while the inhibition of preformed biofilms was assessed against *S. aureus*, *P. mirabilis*, and *P. aeruginosa*. To explore the binding modes and affinity of the investigated silver(I) complexes, a molecular docking study was performed against the LasR transcriptional regulator, while in silico ADMET screening was conducted using the pkCSM online platform. Finally, the evaluation of in vitro immunomodulatory effects of the silver(I) complexes was assessed by measuring cytokine concentrations and performing flow cytometry. **Results:** In vitro and in silico evaluations confirm that the **C2** complex possesses an optimal structural balance of size and hydrophobicity, enabling superior antimicrobial and antibiofilm activity against *P. aeruginosa*. Regarding the immune response, treatment with the **C3** complex decreased the concentrations of the proinflammatory cytokines IL-1β, TNF-α, IL-6, and IL-12, while increasing the concentration of the anti-inflammatory cytokine IL-10 after LPS stimulation. **Conclusions:** Our findings demonstrate that **C2** primarily targets bacterial pathogens, while **C3** exerts potent immunomodulatory and anti-inflammatory effects that differ between innate and acquired, naïve and primed immune system cells. Therefore, investigating the combined or sequential application of **C2** and **C3** may provide a promising strategy for the treatment of chronic biofilm-associated infections.

## 1. Introduction

The use of silver in the treatment of various medical conditions dates back to ancient times. Hippocrates recognized the beneficial effects of silver and utilized preparations based on this metal to treat various skin diseases, primarily ulcers and purulent wounds, in order to prevent infection and accelerate tissue regeneration [1]. Over the centuries, numerous silver-based formulations have been developed for diverse clinical indications. During the 19th century, Dr. James Marion Sims employed silver sutures to close wounds during surgical interventions, which drastically reduced infection rates. Following this success, silver catheters were introduced for the management of urinary tract infections [2,3]. Furthermore, silver nitrate found a vital application in preventing ophthalmia neonatorum in newborns [4]. This widespread expansion of silver preparations lasted until the mid-20th century with the discovery and commercialization of antibiotics. However, as the uncontrolled use of antibiotics has led to a dramatic rise in antimicrobial resistance among numerous bacterial strains, posing a critical global health threat, modern medicine is once again turning toward silver ion-based compounds [5]. The most prominent registered silver preparation globally, silver sulfadiazine, remains widely utilized today as a key drug for the treatment and prevention of infections in severe burns [6].

Literature data indicate that the clinical use of silver in low doses does not induce the severe systemic toxicity characteristic of platinum-group metals. However, prolonged exposure to silver may lead to its accumulation in organs and tissues, potentially triggering the development of argyria, leukopenia, and liver damage [7]. Nevertheless, this relatively favorable safety profile compared to other transition metal ions has strongly encouraged the development of novel coordination compounds utilizing silver(I) ions as central metal ions.

Thiosalicylic acid (TSA) and its various derivatives exhibit a wide spectrum of pharmacological and industrial applications, including fields from cosmetics to medicine [8,9,10,11]. Specifically, studies indicate that thiosalicylate is capable of inhibiting the increase in interstitial calcium in the thymus, as well as suppressing the release of vascular endothelial growth factor (VEGF) induced by thimerosal [12]. Additionally, the TSA derivative farnesylthiosalicylic acid acts as a synthetic mimetic of farnesylcysteine, demonstrating potent activity as an inhibitor of Rat Sarcoma (Ras) protein signaling in transforming cancer cells [13]. In view of these attributes, TSA derivatives represent highly significant, biologically active organic ligands. Furthermore, their coordination potential is remarkably diverse due to the unique combination of ”hard” and ”soft” donor atoms within their structure [14].

Silver exerts its antimicrobial effect only in an ionized state or in the form of a coordination compound, whereas elemental silver does not possess this efficacy. Compared to other transition metals, silver exhibits significantly lower toxicity, with the added advantage that, in appropriate concentrations, it is not considered carcinogenic and has even demonstrated promising anticancer potential. Following the synthesis of silver sulfadiazine in the 1960s as the first combination of silver and sulfonamides, numerous studies have been conducted to synthesize compounds with a broader and safer antibacterial profile and greater efficacy against various pathogenic microorganisms [15,16,17]. Thus, silver complexes with sulfamethoxazole and sulfathiazole were synthesized, demonstrating more potent activity against Gram-negative bacteria than against the tested Gram-positive strains [18]. Recent studies evaluating the antibacterial effect of silver sulfadiazine have confirmed that this complex remains the most effective combination of silver and sulfonamides, and that its application is irreplaceable in the treatment of bacteria that have developed resistance to numerous known antibiotics, primarily methicillin-resistant *S. aureus* (MRSA) [19]. Beyond these specific complexes, silver nanoparticles generally exhibit broad-spectrum antimicrobial activity against numerous bacterial strains with a low risk of resistance. Furthermore, their therapeutic potential extends to nematicidal and anthelmintic properties. Additionally, silver nanoparticles coated with plant biomolecules are capable of scavenging free radicals and exhibit highly pronounced antioxidant activity [20,21,22].

Recent literature highlights the significance of silver coordination compounds with nonsteroidal anti-inflammatory drugs (NSAIDs) [23]. Specifically, the silver–nimesulide complex exhibits significant immunomodulatory activity; through the interaction of silver with thiol groups, it modulates the immune response by inhibiting cyclooxygenase-2 (COX-2) and reducing the levels of crucial pro-inflammatory cytokines, such as tumor necrosis factor alpha (TNF-α) and interleukin-1 beta (IL-1β). This mechanism expands its therapeutic scope and enables a synergistic effect in treating both infection and inflammation [24].

The biofilm matrix protects microorganisms from both host immune responses and antimicrobial agents, thereby promoting persistent infections and significantly complicating their treatment [25]. In addition to conferring enhanced antimicrobial tolerance, biofilms promote chronic inflammation, increased leukocyte infiltration, tissue damage, and delayed wound healing [26]. Consequently, increasing attention has been directed toward multifunctional antimicrobial agents capable of simultaneously targeting microbial pathogens and modulating the host immune response. In this context, antimicrobial coatings containing metal ions have been proposed as an effective strategy for preventing or eradicating biofilms through both direct antimicrobial activity and reprogramming of the local immune microenvironment [27]. Therefore, the development of novel silver(I) complexes capable of simultaneously inhibiting planktonic bacterial growth, preventing biofilm formation, and modulating excessive inflammatory responses represents a promising therapeutic approach [28,29,30]. Such multifunctional compounds could simultaneously reduce the microbial burden and attenuate excessive inflammation, potentially improving therapeutic outcomes while reducing the need for combined antimicrobial and anti-inflammatory therapies [31]. Furthermore, silver complexes attract significant interest due to their highly pronounced antitumor activity, which is exerted through diverse mechanisms of action. Thus, the *bis*(1,10-phenanthroline)-Ag(I) acetate monohydrate complex depolarizes the mitochondrial membrane and stimulates production of reactive oxygen species, thereby inducing cell apoptosis by altering the mitochondrial oxidative phosphorylation pathway. The silver pyridine-2-sulfonate complex exhibits an antitumor effect through the inhibition of topoisomerase I-mediated cleavage of pBR322 DNA, consequently leading to cell death. By disrupting cell membrane integrity, silver–phosphine complexes induce cell cycle arrest in the S and G2/M phases, which ultimately triggers oxidative damage to DNA and mitochondrial membranes [32,33].

As a continuation of our previous research that demonstrated the extensive antitumor activity of novel silver(I) complexes with thiosalicylic acid derivatives [34], the present study evaluates their broader therapeutic potential. The targeted design of the tested complexes with novel structural modifications, achieved by introducing various aliphatic and aromatic moieties, allows a systematic assessment of how these features directly affect the biological response, establishing a clear structure–activity relationship (SAR) that has previously been unavailable for silver complexes with thiosalicylic acid derivative ligands. The main objective of this study was to determine their in vitro antimicrobial efficiency, antibiofilm capacity, and immunomodulatory effects. Additionally, these biological assays were supported by in silico investigations to elucidate the underlying molecular mechanisms and predict the key pharmacological parameters of the compounds.

## 2. Materials and Methods

### 2.1. Test Substances and Chemicals

The methodology of the present study includes biological and computational analyses that follow up on our previously established research. The investigated ligands (S-alkyl derivatives of TSA) and silver(I) complexes were synthesized according to previously published procedures, as detailed in our prior reports [34,35], and their chemical structures are illustrated in Figure 1.

For the in vitro antimicrobial assay, the tested substances were dissolved in 10% dimethyl sulfoxide (DMSO; Acros Organics, Bridgewater, NJ, USA) and further diluted with sterile distilled water. Resazurin was obtained from Alfa Aesar GmbH & Co. (Karlsruhe, Germany). Tetracycline (Sigma Chemicals Co., St. Louis, MO, USA) was dissolved in Mueller–Hinton broth (Torlak, Belgrade, Serbia), while fluconazole (Pfizer, New York, NY, USA) was dissolved in Sabouraud broth (Torlak, Belgrade, Serbia). Based on promising results obtained in our previous study [34], the **C3** complex was chosen for this in vitro immunomodulatory assessment.

### 2.2. In Vitro Antimicrobial Assay

#### 2.2.1. Test Microorganisms and Preparation of Microbial Suspensions

Antimicrobial activity was evaluated against ten microorganisms: nine bacterial strains, including five reference strains (*Staphylococcus aureus* ATCC 25923, *Bacillus subtilis* ATCC 6633, *Proteus mirabilis* ATCC 12453, *Escherichia coli* ATCC 25922, and *Pseudomonas aeruginosa* ATCC 27853), and four clinical isolates (*S. aureus*, *E. coli*, *Salmonella enterica*, and *B. subtilis*), and one fungal strain, *Candida albicans* ATCC 10231. Clinical isolates were provided by the Institute of Public Health, Kragujevac, while reference strains were obtained from the Microbiology Laboratory, Faculty of Science, University of Kragujevac.

Bacterial suspensions were prepared using the direct colony method, with turbidity adjusted to the 0.5 McFarland standard (1.5 × 10^8^ CFU/mL for bacteria and 10^6^ CFU/mL for yeast) using a densitometer (DEN-1, BioSan, Riga, Latvia) [36]. For testing, suspensions were further diluted 1:100 in sterile 0.85% saline.

#### 2.2.2. Microdilution Method

Minimum inhibitory concentrations (MICs) and minimum microbicidal concentrations (MMCs) were determined using the resazurin-based microdilution method [37]. Briefly, 96-well plates were prepared with 100 μL Mueller–Hinton broth (for bacteria) or Sabouraud broth (for fungi) per well. A 100 μL aliquot of the test compound stock solution was added to the first well of each row, followed by twofold serial dilutions to obtain final concentrations ranging from 500 to 0.48 µg/mL [38]. Tetracycline and fluconazole served as positive controls, while 10% DMSO was included as a solvent control and showed no inhibitory effect. Growth and sterility controls were included in each assay.

MIC was defined as the lowest concentration preventing resazurin color change or visible fungal growth. To determine MMC, 10 μL from wells without growth were plated on nutrient agar. The lowest concentration yielding no colony formation after incubation was recorded as the MMC. Antimicrobial assays were performed in three independent biological experiments using freshly and independently prepared microbial inocula. Within each experiment, all tested concentrations were assessed in duplicate. Since identical MIC and MMC values were obtained in all three independent experiments, the results are presented as single representative values.

#### 2.2.3. Determination of Antibiofilm Activity

##### Biofilm Formation

The biofilm-forming ability of *S. aureus* ATCC 25923, *P. mirabilis* ATCC 12453, and *P. aeruginosa* ATCC 27853 was evaluated according to O’Toole et al. [39] with minor modifications. Briefly, 96-well tissue culture microtiter plates (Sarstedt, Germany) were filled with 100 μL Mueller–Hinton broth per well, followed by inoculation with 10 μL of bacterial suspension (1.0 McFarland for Gram-positive and 0.5 McFarland for Gram-negative strains). Plates were incubated at 37 °C for 48 h to promote biofilm formation. Following incubation, wells were washed twice with 200 μL sterile 0.85% saline to remove non-adherent and weakly adherent cells. Biofilms were fixed with 100 μL methanol until complete evaporation, stained with 0.1% (*w*/*v*) crystal violet (Fluka AG, Buchs, Switzerland) for 20 min at room temperature, and washed thoroughly with deionized water followed by 96% ethanol. The optical density (OD) of adherent biofilm cells was measured at 630 nm using a microplate reader (RT-2100C, Rayto, Shenzhen, China). Sterility and nonspecific binding were assessed using broth-only controls. Background absorbance from sterile medium, fixative, and dye was subtracted from all test values. All assays were performed in three independent experiments, each conducted in duplicate.

##### Inhibition of Preformed Biofilms

For inhibition assays, biofilms were first established in 96-well plates by inoculating 100 μL of Mueller–Hinton broth with 10 μL of bacterial suspension (prepared as described above) and incubating at 37 °C for 48 h. After incubation, planktonic cells were carefully removed, and 100 μL of fresh broth containing serial dilutions of the tested silver(I) complexes (500–0.48 µg/mL) was added to each well. Plates were incubated at 37 °C for 24 h. Subsequent washing, fixation, staining, and OD measurement were performed as described for the biofilm formation assay. The biofilm inhibitory concentration (BIC_50_) was defined as the lowest silver(I) complex concentration required to reduce biofilm biomass by 50%, while BIC_90_ represented a 90% reduction [40]. Vancomycin (Lek farmaceutska družba, Ljubljana, Slovenia) and ceftriaxone (Galenika A.D., Belgrade, Serbia) dissolved in nutrient broth were used as reference compounds. Unlike the silver(I) complexes, for which the highest tested concentration was 500 µg/mL, the reference antibiotics were tested over a broader concentration range extending up to 1000 µg/mL. Therefore, BIC_50_ values above 500 µg/mL reported for the reference antibiotics were obtained within the experimentally tested concentration range and did not represent extrapolated values. Controls included broth alone and broth with silver(I) complexes to verify sterility and nonspecific binding. Background absorbance values from controls were subtracted from test readings. Biofilm inhibition assays were performed in three independent experiments, each conducted in duplicate. For each tested concentration, the mean biofilm inhibition values obtained from the independent experiments were used to construct concentration–response curves, and BIC_50_ and BIC_90_ values were estimated by nonlinear regression analysis. When the estimated BIC_50_ or BIC_90_ exceeded the highest tested concentration, the results were reported as >500 µg/mL for the silver(I) complexes and >1000 µg/mL for the reference antibiotics, respectively.

### 2.3. Molecular Docking

#### 2.3.1. Target Protein Preparation

To explore the binding modes and affinity of the investigated silver(I) complexes, a molecular docking study was performed against the LasR transcriptional regulator as the target protein [41]. The crystal structure of the *P. aeruginosa* LasR ligand-binding domain (PDB ID: 2UV0) was downloaded from the Protein Data Bank (http://www.rcsb.org/pdb, accessed on 25 May 2026). Since the structure of LasR is a symmetrical dimer, it was prepared by removing unnecessary chains, water molecules, and the autoinducer as a co-crystallized ligand (*N*-3-oxo-dodecanoyl-*L*-homoserine lactone). Polar hydrogen atoms and Kollman partial charges were subsequently assigned to the target protein using the graphical user interface of the AutoDockTools program (version 1.5.6) [42].

#### 2.3.2. Molecular Structure Preparation and Quantum-Chemical Geometry Optimization

The molecular structures of the investigated silver complexes were initially constructed using ChemDraw Professional 20.0 (PerkinElmer Informatics, Waltham, MA, USA) [43]. The corresponding three-dimensional structures were generated and inspected using Chem3D 20.0 (PerkinElmer Informatics, Waltham, MA, USA) [44] and Avogadro2 Version 2.0.0 [45]. These structures were subsequently used as the starting geometries for quantum-chemical optimization.

The geometries of the silver-containing complexes (**C1**–**C5**) were optimized using the ORCA 6.1.1 quantum-chemical program package [46]. The calculations were performed within the framework of density functional theory (DFT) using the B3LYP exchange-correlation functional combined with Grimme’s D3 dispersion correction with Becke–Johnson damping (B3LYP-D3(BJ)). The RIJCOSX approximation was employed for the hybrid DFT calculations, together with TightSCF convergence criteria and the DefGrid3 numerical integration grid.

A mixed basis-set approach was used for the geometry optimization. The C, H, O, and S atoms were described using the 6-311+G(d,p) basis set, whereas the Ag atoms were treated using the def2-TZVP basis set in conjunction with the corresponding def2 effective core potential (def2-ECP). The use of the effective core potential for Ag accounts for the core electrons of the silver centers while allowing the valence electrons to be described using the def2-TZVP basis set.

The complexes were treated as neutral singlet systems (charge = 0, multiplicity = 1), and no geometrical constraints were imposed during the optimization. The geometry optimizations were continued until the convergence criteria implemented in ORCA 6.1.1 were satisfied. Four CPU cores were used for each calculation, with a maximum memory allocation of 2000 MB per core. The optimized geometries were subsequently used as the input structures for the molecular docking calculations described below.

#### 2.3.3. Molecular Docking Protocols

Molecular docking was performed using AutoDock 4.2 software [42] by fitting the flexible structures of the silver(I) complexes into the rigid structure of the target protein. The docking grid box dimensions were set to 40 × 40 × 40 Å^3^, separated by 0.375 Å, while the grid center coordinates were defined according to the position of the co-crystallized ligand, which acts as an autoinducer (x = 23.016, y = 15.859, z = 80.066).

The Lamarckian Genetic Algorithm was employed under default settings, including a population size of 150, a maximum number of energy evaluations, and generations of 2.5 million and 27,000, respectively, with a mutation rate of 0.02 and a crossover rate of 0.8. We modified the database files AD4.1_bound.dat and AD4_parameters.dat by adding a custom parameter line for the silver atom at the end of each file. The grid energy maps for the silver atom were calculated by adding these atom-specific parameters to the force field grid generation tool (AutoGrid):

atom_par Ag     3.15  0.036   12.000  −0.00110  0.0  0.0  0  −1  −1  1  # Non H-bonding.

Ten docking runs were generated for each ligand as possible binding conformations, while the best-ranked docking poses of the investigated silver(I) complexes were selected for further analysis and visualization using PyMOL 2.4.1 [47].

### 2.4. In Silico ADMET Prediction

To evaluate the pharmacokinetic properties and safety profile of the tested ligands, an in silico ADMET screening was performed using the pkCSM online platform [48]. The ligand structures were converted into canonical SMILES format, and the key parameters of absorption, distribution, metabolism, and organ-specific toxicity were predicted.

### 2.5. Evaluation of In Vitro Immunomodulatory Effects

#### 2.5.1. Splenocytes Isolation

Splenocytes were isolated from the spleens of BALB/c mice according to previously described protocols [49]. After isolation, cell suspensions with viability higher than 95% were seeded in a 96-well microtiter plate at a density of 2 × 10^5^ cells per well. Cells were incubated in Dulbecco’s modified eagle medium (DMEM) (control, untreated group), DMEM with **C3** (0.061 µg/mL), DMEM with Lipopolysaccharide (LPS) (0.5 μg/mL), DMEM with Concanavalin A (ConA) (0.5 μg/mL), DMEM with LPS and **C3**, or ConA and **C3**. Cells were incubated for 24 h under standard conditions.

#### 2.5.2. Cytokine Concentration Measurement

After the incubation period, microtiter plates were centrifuged, and supernatants were collected. According to manufacturer instructions, ELISA assay kits were used to measure cytokine concentrations (R&D Systems, Minneapolis, MN, USA). Concentrations of interleukins (IL), IL-1β, IL-6, IL-12, IL-10, IL-17, TNF-α, and interferon-γ (IFN-γ) were determined and presented as mean ± SEM (standard error of mean).

#### 2.5.3. Flow Cytometry

At the end of the incubation period, cells from all examined groups were harvested and stained for F4/80, CD206, CD3, or CD69. Following incubation required for intracellular staining, which was performed according to a previously published protocol [50], anti-mouse antibodies specific for IL-1β, TNF-α, IL-6, IL-12, IL-10, IFN-γ and IL-17 were used for intracellular cytokine staining. Samples were analyzed using a FACSCalibur flow cytometer (BD Biosciences, San Jose, CA, USA). Data analysis was performed using FlowJo software Version 10.7.2 (Tree Star Inc. Ashland, OR, USA).

#### 2.5.4. Statistical Analysis

MIC and MMC values, which represent discrete endpoints obtained using twofold serial dilutions, were analyzed descriptively. Identical MIC and MMC values were obtained across all three independent experiments, and the modal values are therefore reported. BIC_50_ and BIC_90_ values were estimated from concentration–response curves generated by nonlinear regression using Microsoft Excel. SPSS (version 23.0) was used for data analysis. As not all data were normally distributed according to the Shapiro–Wilk test, differences among groups were analyzed using the Kruskal–Wallis test followed by the Mann–Whitney U test as a post hoc test. Data are presented as mean ± SEM. Differences were considered statistically significant at *p* ˂ 0.05.

## 3. Results and Discussion

### 3.1. Antimicrobial Activity of Investigated Ligands and Silver(I) Complexes

The results of in vitro antimicrobial activities of tested ligands and silver(I) complexes, determined by MICs and MMCs, are shown in Table 1. A total of 10 species of microorganisms were tested, and the results were compared with the influence of tetracycline for bacteria and with fluconazole for fungi (Table 1).

The tested ligands (**L1**–**L5**) generally exhibited weak or no antimicrobial activity, with MIC and MMC values typically exceeding 500 µg/mL against most microorganisms (Table 1). Among them, **L2** and **L5** demonstrated slightly better effects, with MICs of 250 µg/mL against *E. coli* ATCC 25922 (**L2**, **L5**) and 500 µg/mL against clinical *E. coli* and *P. mirabilis* ATCC 12453 (**L1**–**L3**). **L4** also showed activity at 500 µg/mL against *B. subtilis* isolate, *S. aureus* ATCC 25923, and *P. aeruginosa* ATCC 27853. However, in all cases, MIC values remained relatively high, and the corresponding MMC values were equal to or above MICs, indicating limited bactericidal or fungicidal effects.

In contrast, silver complexes (**C1**–**C5**) displayed markedly enhanced antimicrobial activity across all tested microorganisms. MIC values ranged from 3.9 to 62.5 µg/mL for bacteria, with the lowest concentrations recorded against *P. aeruginosa* ATCC 27853 (MIC = 3.9 µg/mL for **C1**–**C4**) and *E. coli* ATCC 25922 (MIC = 7.8 µg/mL for **C2**). These findings are of particular importance, as both *P. aeruginosa* and *E. coli* are clinically relevant pathogens frequently associated with antibiotic resistance. Among the tested compounds, **C5** emerged as the most active, with MICs as low as 7.8 µg/mL against *S. aureus* ATCC 25923 and *P. mirabilis* ATCC 12453, and 15.6 µg/mL against *E. coli* strains, confirming its broad-spectrum potential.

The complexes also demonstrated activity against Gram-positive bacteria; however, MIC values were generally higher compared to Gram-negative strains. For example, **C2** and **C5** inhibited *S. aureus* ATCC 25923 at 31.25 µg/mL and 7.8 µg/mL, respectively. This variability may reflect structural differences in bacterial cell envelopes, with Gram-negative species being more susceptible to silver-based compounds due to outer membrane interactions and increased silver ion uptake.

Moderate antifungal activity was observed against *C. albicans* ATCC 10231, with MIC values ranging from 31.25 µg/mL (**C5**) to 250 µg/mL (**C4**). Although less potent than the antibacterial effects, these findings suggest that silver complexes may still exert inhibitory action against eukaryotic microorganisms.

The present study clearly demonstrates that coordination of the investigated ligands with Ag(I) markedly enhanced their antimicrobial activity. Whereas the free ligands exhibited little or no inhibitory effect at the tested concentrations, complex formation resulted in a substantial reduction in MIC values, confirming that silver coordination is essential for biological activity. These findings are consistent with previous reports showing that complexation not only stabilizes Ag(I) ions but may also improve their antimicrobial efficacy by modulating their physicochemical properties and interactions with microbial cells [51]. Silver has been recognized as a broad-spectrum antimicrobial agent for centuries and has been used in various forms, including metallic silver, inorganic salts, and coordination compounds [52]. Although the widespread introduction of antibiotics reduced its clinical use, silver-based therapeutics have regained considerable attention because of their activity against multidrug-resistant microorganisms, broad antimicrobial spectrum, and relatively low toxicity [53]. Nevertheless, despite their promising in vitro activity, many silver complexes exhibit limited in vivo efficacy due to rapid systemic clearance, a common limitation of low-molecular-weight compounds. Consequently, alternative delivery approaches, including encapsulation into biodegradable nanocarriers, have been proposed to prolong circulation time, improve bioavailability, and enhance therapeutic efficacy [53,54,55].

Among clinically used silver-containing agents, silver sulfadiazine (SSD) remains the gold standard for the topical treatment and prevention of wound-associated infections, owing to its broad-spectrum antibacterial and antifungal activity, favorable safety profile, and extensive clinical use [56,57,58]. Hamilton-Miller et al. [59] reported MIC values for SSD generally ranging from 16 to 64 µg/mL against susceptible bacterial species. Although SSD was not included as a reference compound in the present study, these findings are in agreement with numerous recent studies demonstrating that Ag(I) coordination compounds containing salicylate–metronidazole, benzimidazole, econazole, or miconazole ligands consistently exhibit potent activity against clinically relevant Gram-positive, Gram-negative, and fungal pathogens [60,61,62,63,64]. These studies indicate that ligand selection strongly influences the antimicrobial spectrum and potency of silver(I) complexes.

The antimicrobial activity of silver-containing compounds depends not only on the amount of released Ag^+^ ions but also on their chemical form, stability, and interaction with bacterial cells. In agreement with this concept, ref. [65] demonstrated that silver nitrate, despite releasing the highest concentration of free Ag^+^ ions, exhibited lower antimicrobial activity against *P. aeruginosa* and *S. aureus* than electrolyzed silver and silver nanoparticles. These findings suggest that the biological activity of silver-based agents cannot be explained solely by silver ion availability. Instead, the physicochemical characteristics of the silver species, including their stability, cellular uptake, controlled silver ion release, and interactions with bacterial membranes, play a crucial role in determining antimicrobial efficacy. The differences observed among the synthesized silver(I) complexes in the present study may be attributed to the influence of ligand coordination on these properties.

The MIC values obtained in the present study fall within the range reported for several investigated silver-based antimicrobials. However, direct extrapolation of in vitro antimicrobial concentrations to clinical application should be made with caution because local silver concentrations at the site of infection depend on the formulation, release kinetics, tissue penetration, protein binding, and wound exudate composition [66,67]. This consideration is particularly relevant for topical silver formulations, where local drug concentrations are substantially higher than those achieved following systemic administration. Furthermore, although silver-containing compounds are generally considered to have a lower propensity to promote bacterial resistance than conventional antibiotics, their clinical translation requires comprehensive evaluation of cytotoxicity, pharmacokinetics, biodistribution, and long-term safety [68]. Nevertheless, the favorable in vitro antimicrobial activity demonstrated in the present study, together with accumulating evidence from structurally related Ag(I) complexes, supports the further preclinical development of these compounds as promising silver-based antimicrobial agents.

### 3.2. Inhibition of Preformed Bacterial Biofilms

Biofilms were quantified by measuring the absorbance of stained biofilms at 630 nm with an ELISA plate reader. The results indicated that *P. aeruginosa* ATCC 27853, *P. mirabilis* ATCC 12453 and *S. aureus* ATCC 25923 could form biofilms better than other bacteria studied. The absorbance values were 1.5 for *P. aeruginosa* ATCC 27853, 0.47 for *P. mirabilis* ATCC 12453 and 0.54 for *S. aureus* ATCC 25923.

The antibiofilm activity of the synthesized silver(I) complexes varied among the tested bacterial strains (Table 2). Overall, none of the complexes achieved a 90% reduction in biofilm biomass within the tested concentration range (BIC_90_ values > 500 µg/mL). Among the tested compounds, **C2** exhibited the lowest BIC_50_ value (75 µg/mL) against *P. aeruginosa* ATCC 27853, indicating stronger antibiofilm potential compared to other complexes. Similarly, **C1** displayed notable activity against *S. aureus* ATCC 25923 with a BIC_50_ of 128 µg/mL, while **C3** demonstrated moderate effects against *P. mirabilis* ATCC 12453 (BIC_50_ = 332 µg/mL). By contrast, **C5** did not show relevant antibiofilm activity across the tested strains, with BIC_50_ values exceeding 362 µg/mL. These findings highlight a strain-dependent response to silver(I) complex treatment, with *P. aeruginosa* biofilms being the most susceptible, consistent with its reported sensitivity to silver-based antimicrobials. However, the inability of the complexes to achieve 90% biofilm biomass reduction within the tested concentration range suggests that higher concentrations, structural optimization, or combinatorial approaches may be necessary to enhance efficacy.

The activity of the synthesized silver(I) complexes against bacterial biofilms is of particular pharmaceutical relevance, considering that biofilm-associated infections on chronic wounds, urinary catheters, and implanted medical devices remain among the most difficult infections to eradicate using conventional antimicrobial therapy. The increased tolerance of biofilm-associated microorganisms arises from the protective extracellular polymeric matrix, reduced metabolic activity, and limited penetration of antimicrobial agents, all of which substantially diminish the efficacy of standard antibiotics. Consequently, the development of new silver-based compounds capable of interfering with biofilm development or disrupting established biofilms represents an important therapeutic objective.

Silver(I) complexes have attracted increasing attention as potential antibiofilm agents because of their broad-spectrum antimicrobial activity, relatively low toxicity, and the structural versatility provided by coordination chemistry, which enables optimization of their biological properties [53]. Most published studies have focused on the ability of silver(I) complexes to inhibit bacterial adhesion or prevent the early stages of biofilm formation. For example, Saleh et al. [69] demonstrated that Ag(I) complexed polyesters based on 2,6-pyridinedicarboxylate effectively inhibited biofilm formation by *P. aeruginosa*, while several other studies reported similar preventive effects against biofilm development [69,70,71,72,73,74]. Likewise, Contini et al. [60] showed that silver(I) salicylate–metronidazole complexes significantly reduced biofilm biomass of *E. coli*, *P. aeruginosa*, *S. aureus*, and *S. epidermidis* when applied at sub-MIC concentrations during biofilm development. Similarly, Şahin et al. demonstrated that a silver(I) complex containing an N-alkylbenzimidazole derivative achieved 67.72% inhibition of *P. aeruginosa* biofilm formation at a concentration of 3 µmol/L [61].

In contrast to these studies, the present work evaluated the activity of silver(I) complexes against preformed bacterial biofilms, which represent a considerably more challenging therapeutic target because of their mature architecture and increased antimicrobial tolerance. To the best of our knowledge, systematic investigations addressing the activity of silver(I) complexes with thiosalicylic acid against established biofilms remain scarce. Although complete biofilm eradication was not achieved within the tested concentration range, the observed reduction in mature biofilm biomass, particularly in *P. aeruginosa*, demonstrates that these complexes retain activity even after biofilm maturation. These findings further support the potential of silver(I) coordination compounds as promising candidates for the development of next-generation antibiofilm therapeutics.

### 3.3. Optimized Molecular Structures and Quantum-Chemical Results

The optimized geometries of complexes **C1**–**C5** were subsequently analyzed in terms of selected structural parameters, including Ag-Ag and Ag-donor atom distances. The calculated structural parameters are summarized in Table 3.

The optimized geometries of the investigated binuclear silver(I) complexes (**C1**–**C5**) are presented in Figure 2 and reveal relatively consistent structural parameters across the series (Table 3). The Ag-Ag distances ranged from 2.7970 to 3.1276 Å, with the shortest distance observed for **C1** (2.7970 Å) and the longest for **C2** (3.1276 Å). In **C1** and **C3**–**C5**, the Ag-Ag separations were relatively similar, ranging from 2.7970 to 2.8529 Å, whereas **C2** exhibited a noticeably larger Ag-Ag distance. The Ag-O bond lengths were generally within the range expected for Ag-O coordination, varying from 2.1245 to 2.3540 Å. Complexes **C1**, **C3**, and **C4** showed particularly narrow ranges of Ag-O distances, indicating relatively similar coordination environments around the two silver centers, while **C2** and **C5** displayed somewhat greater differences between the individual Ag-O distances. The calculated final energies of the optimized structures ranged from −2009.2343 to −2469.5407 Eh, reflecting the different molecular compositions and overall numbers of electrons in the respective complexes rather than providing a direct measure of relative stability between **C1**–**C5**. Overall, the optimized structures retained the characteristic binuclear silver arrangement, with Ag-O coordination interactions preserved throughout the series, as also evident from the optimized molecular structures shown in Figure 2.

### 3.4. Molecular Docking Studies

Since the investigated silver(I) complexes exhibited the highest antibacterial sensitivity against *P. aeruginosa*, along with notable antibiofilm activity, molecular docking studies were performed on LasR, a key transcriptional regulator involved in quorum sensing, biofilm formation, and virulence in this pathogen.

In order to correctly interpret the obtained molecular docking results, a validation procedure was carried out by comparing the spatial orientation of the native co-crystallized ligand with its docked conformation. The result of the validation procedure is expressed as the RMSD (Root Mean Square Deviation) parameter. In this study, the calculated RMSD value was 0.899 Å, which successfully validates the molecular docking protocol (Figure 3).

The molecular docking analysis revealed favorable binding affinities for all tested compounds, with complex **C2** displaying the lowest binding free energy (−10.34 kcal/mol). Interestingly, the **C5** complex also showed a high binding affinity of −9.22 kcal/mol, which is in contrast to its poor antibiofilm efficacy against *P. aeruginosa* (Table 4).

Complex **C1** establishes a significant number of interactions within the active site of the target protein, with the presence of two key hydrogen bonds substantially contributing to the stability of the complex. Specifically, the carboxyl oxygen atom and the sulfur atom of the TSA ligand form two conventional hydrogen bonds with Thr75 at distances of 2.99 Å and 2.97 Å, respectively. Furthermore, residues Trp88 and Phe101 engage in π-π stacked and π-π T-shaped interactions, respectively. Additionally, Val76, Cys79, Leu125, Tyr47, Tyr93, Ala50, Ala127, and Ala105 further stabilize the system through alkyl and π-alkyl hydrophobic interactions.

A very similar interaction pattern is observed for **C2**. However, compared to **C1**, the lone pair of the TSA sulfur atom participates in the formation of an additional hydrogen bond with the Thr115 residue. Furthermore, Tyr64 engages in a π-π stacked interaction, further stabilizing the complex within the binding pocket (Figure 4). The docked complex is bound to the native ligand-binding pocket of the LasR receptor and establishes interactions with key amino acid residues involved in the binding of the natural autoinducer, *N*-3-oxo-dodecanoyl-*L*-homoserine lactone.

The docking results indicate that the binding free energy of complex **C3** is very close to that of complex **C2**, which is further supported by their similar interaction profiles. Specifically, the lone electron pair of the carbonyl oxygen of complex **C3** forms a hydrogen bond with the Ser129 residue, with a bond length of 2.83 Å. At the same time, the other hydrogen bonds previously observed for complexes **C1** and **C2** remain preserved. The network of hydrophobic interactions, predominantly of the π-alkyl type, includes contacts with amino acid residues Tyr56, Trp60, Tyr64, Val76, Leu125, and Ala105. Compared to complex **C1**, the comparable free energy of binding and interaction profile observed for complex **C4** suggests a very similar binding mode within the active site of the transcription factor.

The bulky structure of **C5**, including the presence of multiple aromatic rings, contributes to its increased rigidity, which may prevent the complex from adopting a favorable orientation within the LasR active site. The residues Thr75, Thr115 and Ser129 participate in the formation of conventional hydrogen bonds, while the interactions established by this compound are predominantly hydrophobic in nature, including π-π stacking interaction with Tyr64, a π-π T-shaped interaction with Tyr47, and an amide-π stacking interaction with Gly126. In addition, the benzyl groups interact with Trp88, Phe101, Ile52, Val76, Leu125, Ala50, and Ala70 through multiple π-alkyl hydrophobic interactions.

Among the tested complexes, the complex with the ethyl derivative of TSA (**C2**) demonstrated the highest antibacterial efficacy against *P. aeruginosa* in both in vitro assays and molecular docking simulations against LasR, a key transcriptional regulator involved in quorum sensing. This highlights a crucial balance between lipophilicity and steric requirements within the LasR binding pocket. While the methyl group is likely too small to form optimal hydrophobic interactions, longer alkyl chains (propyl and butyl) and the bulky benzyl group introduce excessive steric hindrance, preventing proper fit into the receptor pocket. Furthermore, larger and highly lipophilic molecules may encounter barriers entering *P. aeruginosa* cells, potentially due to outer membrane constraints or bacterial efflux mechanisms. Therefore, the structural features of the **C2** complex, balancing size and hydrophobicity, might favorably influence its interaction with the bacterial membrane and support its binding to the LasR target site.

The tested silver(I) complexes showed a strong effect against *P. aeruginosa*, suggesting that the so-called “zombie effect” may potentially contribute to the observed antimicrobial activity. In this mechanism, bacteria killed by silver act as a reservoir for the prolonged release of silver ions, which continuously eliminate the remaining viable population. This prolonged biocidal action is driven by Le Chatelier’s principle, as the silver species are not deactivated during the initial killing process and remain active for repeated antibacterial cycles [33,75].

In the study conducted by Damena and co-workers [76], zinc(II), copper(II), and nickel(II) complexes were investigated against the same target, where very similar binding patterns and the same types of interactions were observed. However, less favorable binding energies were reported compared to those obtained for our investigated complexes.

This in silico approach and the results obtained cannot provide a definitive confirmation of the mechanism of action of the synthesized silver(I) complexes, which represents a limitation of the present study. Experimental validation is necessary for a more detailed analysis of their action on this key transcriptional regulator involved in quorum sensing.

### 3.5. In Silico ADMET Profiling

The gradual release of silver(I) ions significantly contributes to the biological activity of the investigated complexes. In fact, a rapid increase in silver(I) ion concentration leads to interaction with chloride ions from body fluids, resulting in the formation of poorly soluble salts that lack biological activity. Therefore, the nature and type of the ligand are essential for the manifestation of the therapeutic effect, given that they serve as carriers for silver(I) ions, the primary source of biological activity. Accordingly, an in silico assessment was conducted to predict the complete pharmacokinetic behavior and toxicity profiles of the investigated ligands (Table 5) [40].

For oral drug administration, intestinal absorption is of paramount importance. This parameter is essential for predicting oral bioavailability, which is a prerequisite for biological activity. The in silico prediction results estimate that the absorption percentage for all tested ligands exceeds 92%, suggesting a high rate of human intestinal absorption and pointing toward a potential for the oral administration of these compounds. Notwithstanding a high absorption rate, the presence of P-glycoprotein (P-gp) as a transmembrane efflux transporter can significantly limit drug concentration in the systemic circulation. The pkCSM platform utilizes a robust, curated dataset to evaluate P-gp interactions. Our prediction reveals that, with the sole exception of **L4**, none of the investigated ligands act as P-gp substrates. Furthermore, blood–brain barrier (BBB) permeability values (logBB) ranging between 0.004 and 0.146 indicate moderate barrier crossing and central nervous system (CNS) penetration. This could represent a potential advantage, after experimental confirmation, for the treatment of bacterial infections of the CNS. Conversely, their moderate penetration through the stratum corneum opens up possibilities for topical application in dermatological formulations to treat superficial wounds and infections. However, all of the above in silico parameters represent only predicted pharmacokinetic properties and preliminary computational insights, which serve as guidelines for future research and require experimental confirmation to definitively demonstrate clinical potential.

Beyond absorption and distribution, the structural role of ligands remains central to the metabolic stability and safety profile of the coordination complexes [77,78]. From a pharmacokinetic perspective, rapid metabolic degradation of the ligand can prompt premature dissociation of the complex, leading to an uncontrolled systemic release of silver ions, which may induce various toxic effects.

To address this, the pkCSM in silico predictor was utilized to assess whether the tested ligands act as substrates or inhibitors of major cytochrome P450 isoforms (CYP3A4, CYP2D6, and CYP2C9). The results indicate that the ligands possess no affinity toward these metabolic enzymes, which is highly beneficial for avoiding adverse metabolic clearance and drug–drug interactions (DDIs). The final step of the in silico evaluation involved safety screening focused on genotoxicity and cardiotoxicity. The in silico Ames mutagenicity prediction suggests that the tested ligands lack mutagenic potential, representing a promising initial indicator for future experimental validation to evaluate safety for potential medical applications. Furthermore, extensive literature highlights that cardiovascular toxicity remains a leading cause of drug attrition in preclinical development [79]. The primary drivers of this failure are lethal arrhythmias characterized by QT interval prolongation, induced by the inhibition of cardiac potassium channels encoded by the human gene hERG. Since all investigated ligands were predicted to be non-inhibitors of both hERG I and hERG II in the pkCSM model, they demonstrated an encouraging theoretical cardiotoxicity profile, suggesting their potential for further development as silver-based therapeutic candidates. However, as these in silico parameters primarily reflect the behavior of the free ligands, they cannot directly determine the definitive pharmacokinetic or safety profile of the corresponding silver(I) complexes.

### 3.6. In Vitro Immunomodulatory Effects of ***C3*** Complex

Although in our previous study **C1**–**C5** complexes demonstrated promising cyto-toxic activity, the **C3** complex showed a better selectivity index than cisplatin in the 4T1 cell line and reduced tumor growth in an in vivo murine 4T1 breast cancer model [34]. Further insights into the possible mechanism of action and the potential safety profile of **C3** were also obtained [34]. Based on these findings, **C3** was selected for ex vivo immunomodulatory testing. The antitumor and immunomodulatory potential of the other synthesized complexes remains to be investigated in future studies. In order to evaluate the immunomodulatory effects of **C3**, splenocytes were isolated from the spleens of BALB/c mice according to a previously published protocol [49]. We first evaluated cytokine production by splenocytes after LPS and **C3** complex treatment. LPS is a ligand for Toll-like receptor 4 on innate immune cells [80]. This interaction leads to stimulation of macrophages and monocytes and initiation of signaling pathways that, among other things, upregulate production of proinflammatory mediators [80,81]. As illustrated in Figure 5, concentrations of proinflammatory cytokines IL-1β (Figure 5A), IL-6 (Figure 5C), and anti-inflammatory IL-10 (Figure 5E) were significantly higher in cell supernatants after LPS treatment compared to untreated splenocytes. Treatment with **C3** complex significantly decreased concentrations of TNF-α (Figure 5B) and IL-12 (Figure 5D) compared to untreated splenocytes. After cotreatment with the **C3** complex and LPS, a significant reduction in concentrations of IL-1β (Figure 5A), TNF-α (Figure 5B), IL-6 (Figure 5C), and IL-12 (Figure 5D) was observed compared to LPS-treated splenocytes. Further, the **C3** complex and LPS cotreatment significantly increased the concentration of IL-10 (Figure 5E) in cell supernatants compared to LPS-treated cells. These findings indicate that the **C3** complex could modulate LPS-induced activation of innate immune cells by inhibiting production of proinflammatory cytokines and stimulating production of anti-inflammatory IL-10.

In order to confirm these findings, the functional phenotype of macrophages after LPS treatment, **C3** complex treatment, and **C3** complex and LPS cotreatment were analyzed by flow cytometry (Figure 6). As splenocytes were previously isolated from the spleen of BALB/c mice, the F4/80 molecule, a known surface marker for murine macrophages [82], was used in order to identify splenic macrophages, one of the most important members of innate immune response. As expected, after LPS treatment, the percentages of IL-1β^+^ (Figure 6A), TNF-α^+^ (Figure 6B), IL-6^+^ (Figure 6C), IL-12^+^ (Figure 6D), and IL-10^+^ (Figure 6E) F4/80^+^ macrophages were significantly higher compared to the untreated group. These results are in line with the increased concentrations of cytokines measured in supernatants (Figure 5). **C3** complex treatment increased percentages of IL-1β^+^ (Figure 6A), TNF-α^+^ (Figure 6B), IL-6^+^ (Figure 6C), and IL-10^+^ (Figure 6E) F4/80^+^ macrophages compared to untreated F4/80^+^ cells. Interestingly, **C3** complex treatment reduced the concentration of TNF-α in splenocyte supernatants (Figure 5B). The percentage of TNF-α^+^F4/80^+^ cells increased following **C3** complex treatment, while TNF-α supernatant concentrations after **C3** complex treatment were reduced compared to the untreated group. Although macrophages are the main source of TNF-α in splenocyte cultures, other cells such as lymphocytes, dendritic cells, NK, and NKT cells could produce TNF-α [83,84,85], suggesting that **C3** complex treatment may reduce the production of TNF-α by other splenic cell populations. Furthermore, **C3** and LPS cotreatment significantly decreased the percentages of IL-1β^+^ (Figure 6A), TNF-α^+^ (Figure 6B), and IL-12^+^ (Figure 6D) F4/80^+^ macrophages compared to LPS-treated F4/80^+^ macrophages. This is in line with the previously observed reduction in concentrations of IL-1β, TNF-α and IL-12 following **C3** complex and LPS cotreatment (Figure 5). The percentage of IL-10^+^ F4/80 macrophages following **C3** complex and LPS cotreatment was higher compared to the LPS-treated group (Figure 6E), supporting the previous finding of increased IL-10 concentration in the supernatant of **C3** and LPS cotreated splenocytes (Figure 5). CD206 is a transmembrane receptor and a surface marker for alternatively activated murine M2 macrophages [86]. Higher expression of CD206 is usually associated with an anti-inflammatory response, primarily induced by IL-4 and IL-13 [87]. On the other hand, as LPS stimulates proinflammatory M1 macrophages, no differences in the percentage of CD206^+^ F4/80^+^ macrophages were observed between medium-treated and LPS-treated groups, as expected (Figure 6F). **C3** complex treatment, as well as **C3** complex and LPS cotreatment, increased the percentage of CD206^+^ F4/80^+^ macrophages compared to the medium-treated and LPS-treated groups but without statistical significance (Figure 6F). **C3** treatment under basal conditions increased the percentages of IL-1β^+^, TNF-α^+^, IL-6^+^, and IL-10^+^ F4/80^+^ macrophages compared with the medium group. Interestingly, **C3** and LPS cotreatment decreased the percentages of IL-1β^+^, IL-12^+^, and TNF-α^+^ F4/80^+^ macrophages compared with LPS treatment alone. These findings suggest that the immunomodulatory effects of **C3** may depend on the activation state of macrophages. According to these data, it seems that the **C3** complex differently affects macrophages under basal and LPS-stimulated conditions, while attenuating the LPS-induced proinflammatory response.

Further, we analyzed the immunomodulatory effects of the **C3** complex on Concanavalin A-stimulated splenocytes. Within the isolated splenocytes, ConA stimulates proliferation and activation of T-lymphocytes [88]. ConA treatment upregulated the production of TNF-α and IL-17 compared to the medium-treated group (Figure 7B,D). The increase in TNF-α and IL-17 concentrations demonstrates the induction of Th1 and Th17 immune responses. Measurement after **C3** complex treatment showed an increase in TNF-α (Figure 7B) and a decrease in IFN-γ (Figure 7C) compared to medium-treated splenocytes. Further, **C3** complex and ConA cotreatment significantly decreased the concentration of IL-1β (Figure 7A) and IL-17 (Figure 7D) compared to ConA-treated splenocytes. Decreased levels of TNF-α (Figure 7B) and IFN-γ (Figure 7C) after **C3** complex and ConA cotreatment were also noted; however, these effects did not reach statistical significance. These findings indicate that **C3** complex may have immunomodulatory effects, attenuating the pro-inflammatory response under ConA-stimulated conditions in ex vivo splenocyte assays.

Further, given the heterogeneous nature of splenocytes, the next step was to select the T-lymphocyte population using CD3 and analyze expression of several crucial molecules by flow cytometry. Firstly, expression of CD69, an early marker of lymphocyte activation, was investigated [89]. As expected, ConA treatment enhanced the percentage of CD69^+^ (Figure 8A), IL-1β^+^ (Figure 8B), TNF-α^+^ (Figure 8C), IFN-γ^+^ (Figure 8D) and IL-17^+^ (Figure 8E) CD3^+^ splenocytes in comparison to the medium-treated group. In the absence of stimulation, treatment with **C3** complex significantly increased the percentage of IL-1β^+^CD3^+^ splenocytes compared to the medium-treated group (Figure 8B). Beyond its established role as a proinflammatory cytokine, IL-1β is able to modulate activation and proliferation of T cells following antigenic/mitogenic signals [90,91], indicating that **C3** complex could regulate immune response depending on the conditions. Following **C3** complex and ConA cotreatment, percentages of CD69^+^ (Figure 8A), TNF-α^+^ (Figure 8C), and IL-17^+^ (Figure 8E) CD3^+^ splenocytes were significantly decreased compared to the ConA-treated group. Given that CD69 is an early activation marker, CD69 downregulation indicates that **C3** could affect early T-cell activation [89]. Further, these cells produce fewer proinflammatory cytokines such as TNF-α and IL-17. These findings are in line with our previous results, which showed decreased concentration of IL-17 in the splenocyte supernatants after **C3** complex and ConA cotreatment (Figure 8). Moreover, this cotreatment increased the percentage of IL-10^+^CD3^+^ splenocytes compared to the ConA-treated group but without statistical significance (Figure 8F), indicating a trend toward suppressing the immune response. These results suggest that immunomodulatory effects of **C3** may differ depending on the activation state of T cells. Under basal conditions, **C3** increased the percentage of IL-1β^+^CD3^+^ splenocytes. However, under ConA-stimulated conditions, **C3** reduced the percentage of CD69^+^, TNF-α^+^, and IL-17^+^CD3^+^ splenocytes compared to the ConA-treated group, indicating attenuation of T-cell activation and pro-inflammatory response. Taken together, these findings suggest a potential immunomodulatory and anti-inflammatory activity of **C3** under the tested conditions.

## 4. Conclusions

The present study demonstrates that the investigated silver(I) complexes exhibit distinct and complementary biological activities. Both in vitro and in silico analyses identified the **C2** complex as the most potent antimicrobial and antibiofilm agent against *P. aeruginosa*. Its favorable biological activity was associated with an optimal balance of molecular size and hydrophobicity, while molecular docking suggested a potential interaction with the LasR quorum-sensing regulator that may contribute to the observed antibacterial effects. In parallel, the **C3** complex might have immunomodulatory effects. It seems that the **C3** complex differently affects macrophages and T cells under basal and stimulated conditions, while attenuating the LPS/ConA-induced proinflammatory response.

Future studies should evaluate the stability, pharmacokinetics, biocompatibility, and in vivo therapeutic efficacy of these complexes to further define their translational potential. In addition, investigating the combined or sequential application of **C2** and **C3** may provide a promising strategy for the treatment of chronic biofilm-associated infections, where simultaneous bacterial eradication and modulation of excessive inflammation are essential for successful therapeutic outcomes.

## Figures and Tables

**Figure 1:**
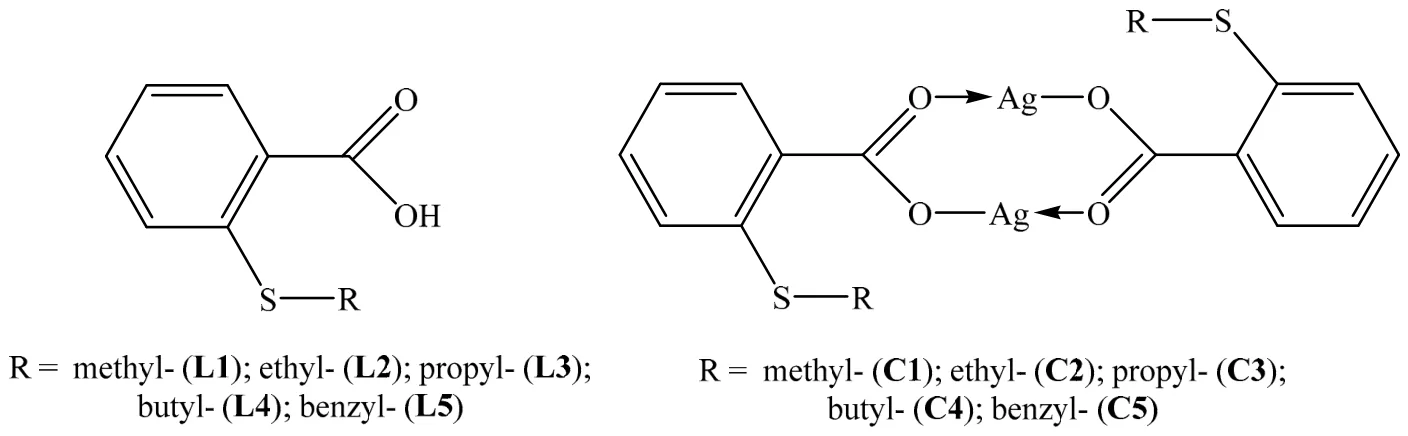
Chemical structures of investigated compounds.

**Figure 2:**
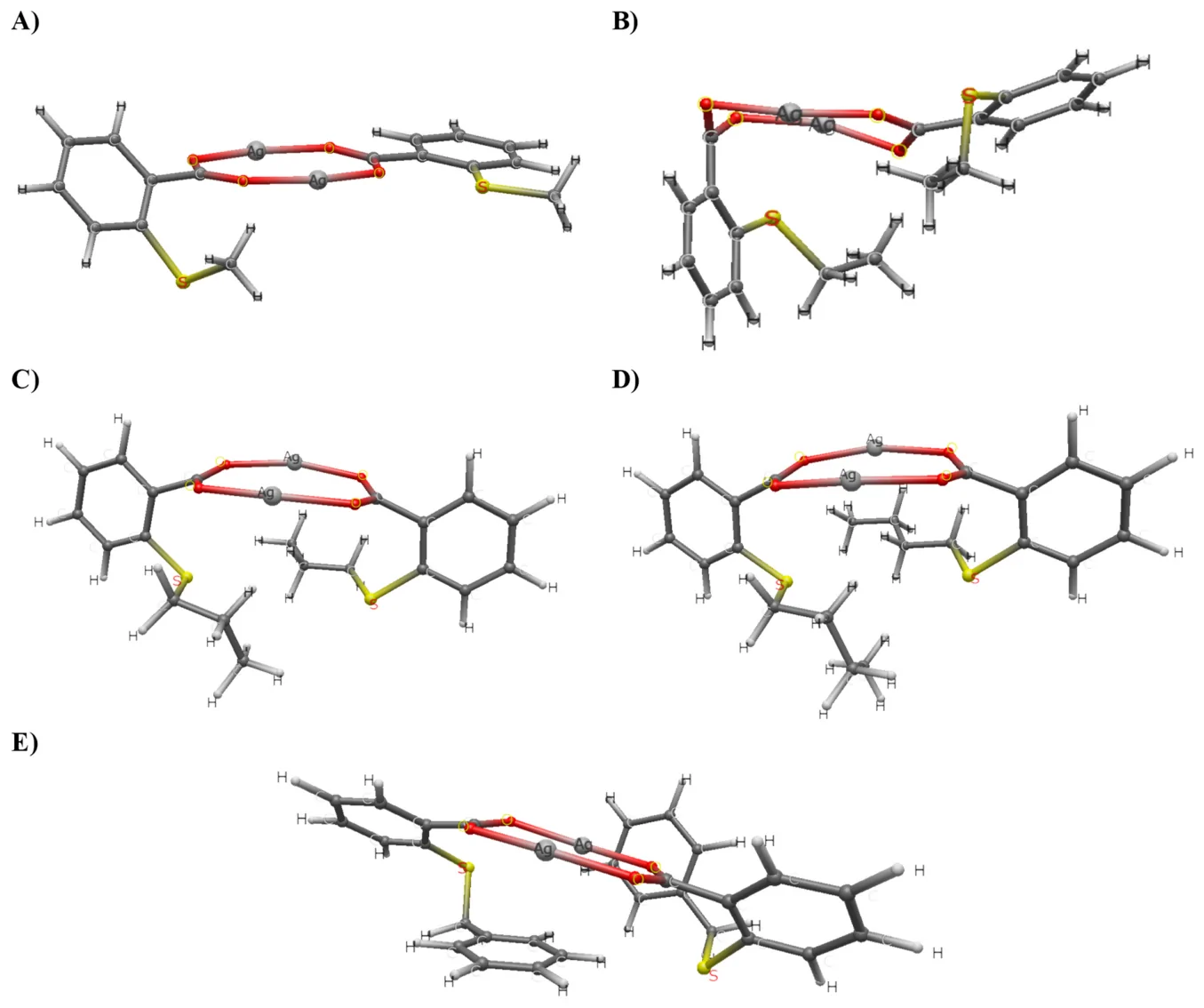
Optimized molecular structures of the investigated binuclear silver(I) complexes **C1**–**C5** (**A**–**E**).

**Figure 3:**
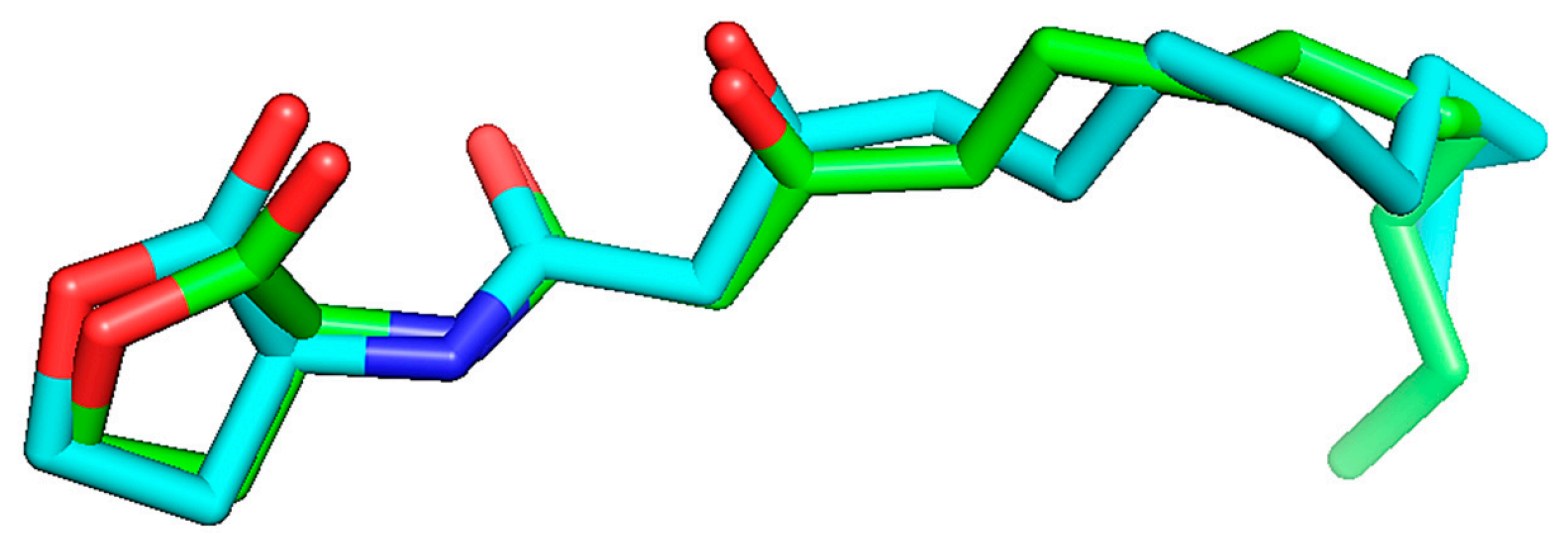
Molecular overlapping of the crystallographic (green) and re-docked (cyan) conformations of the co-crystallized ligand within the LasR ligand-binding domain.

**Figure 4:**
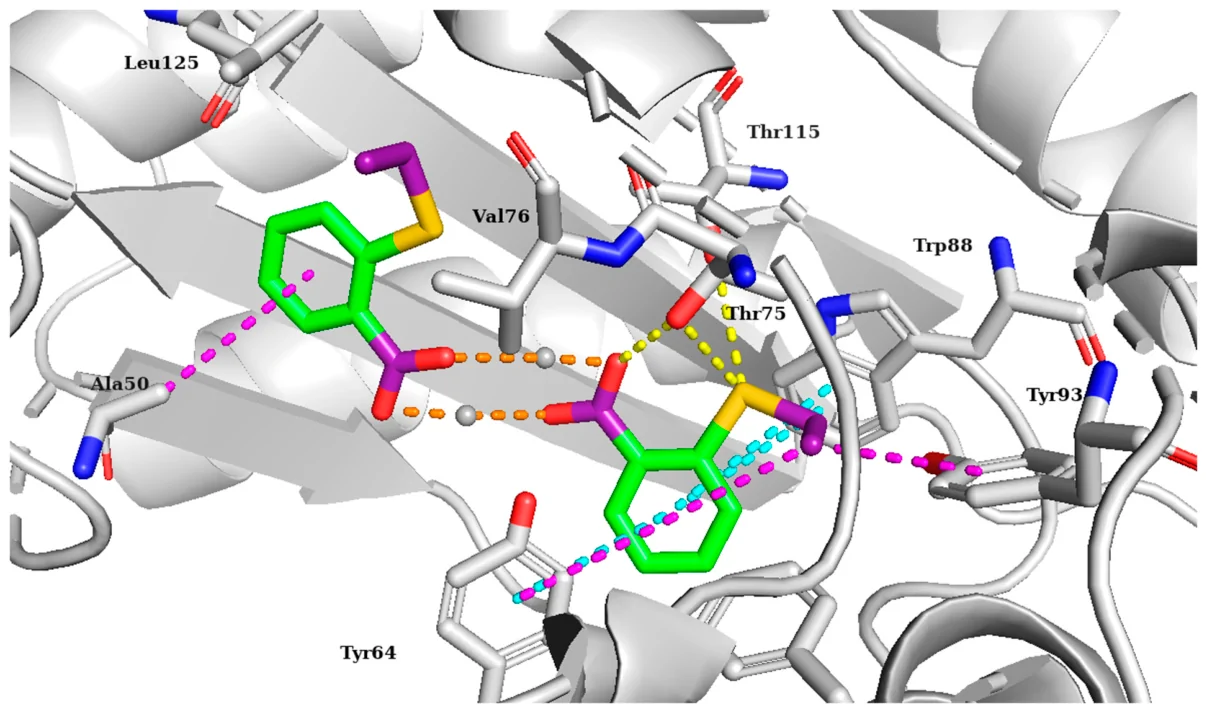
Predicted binding mode of complex **C2** within the active site of LasR (PDB ID: 2UV0). The 3D molecular docking model shows conventional hydrogen bonds represented as yellow dotted lines, π-π stacking interactions as cyan dotted lines, and alkyl and π-alkyl hydrophobic interactions as magenta dotted lines.

**Figure 5:**
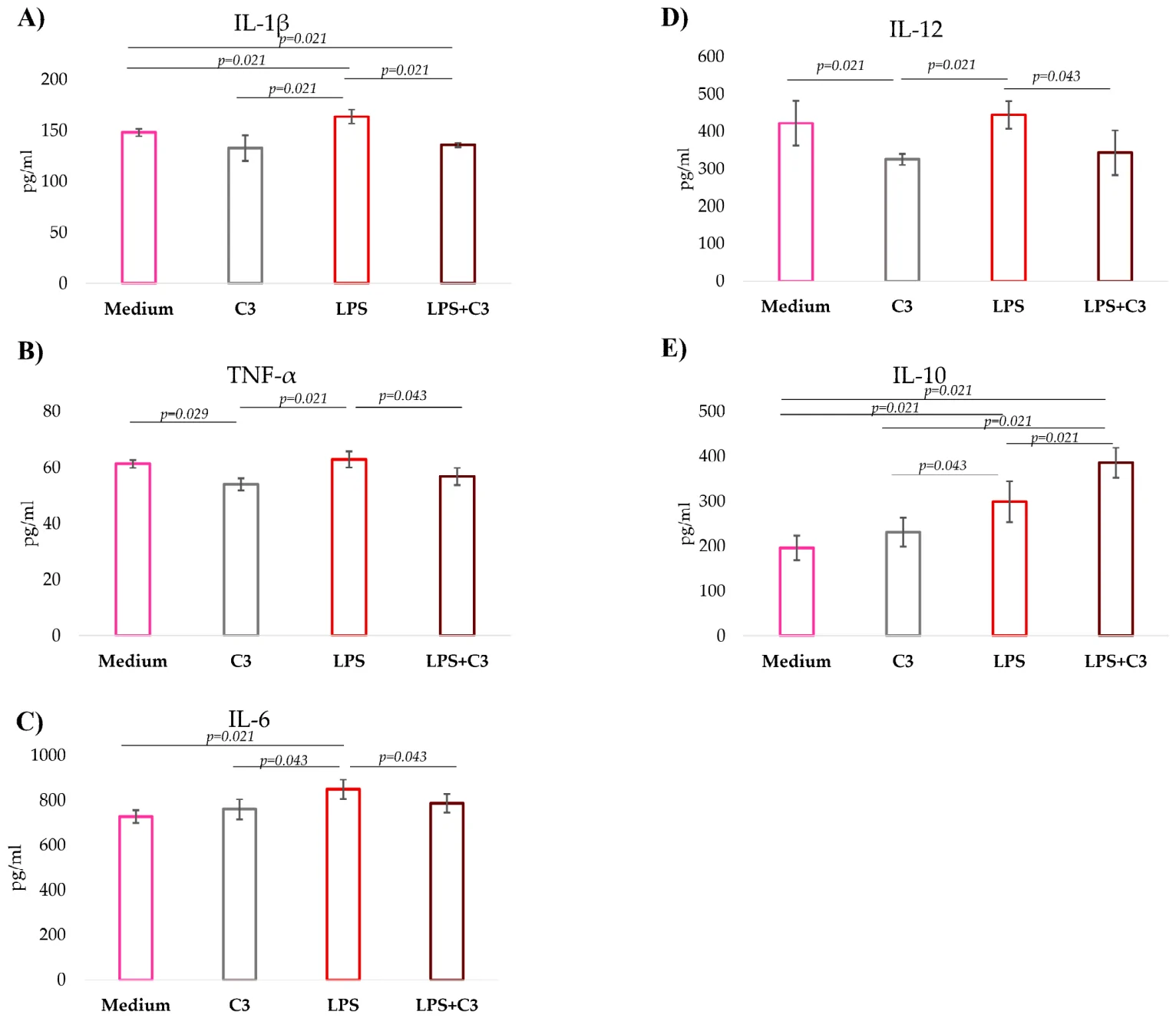
The supernatant splenocyte concentrations of IL-1β (**A**), TNF-α (**B**), IL-6 (**C**), IL-12 (**D**), and IL-10 (**E**) were measured after LPS stimulation or/and **C3** complex treatment. ELISA assay was used to determine cytokine concentrations. Data are presented as mean ± SEM of five mice per group and are representative of three separate experiments. Statistical significance was determined by Kruskal–Wallis test followed by Mann–Whitney U test.

**Figure 6:**
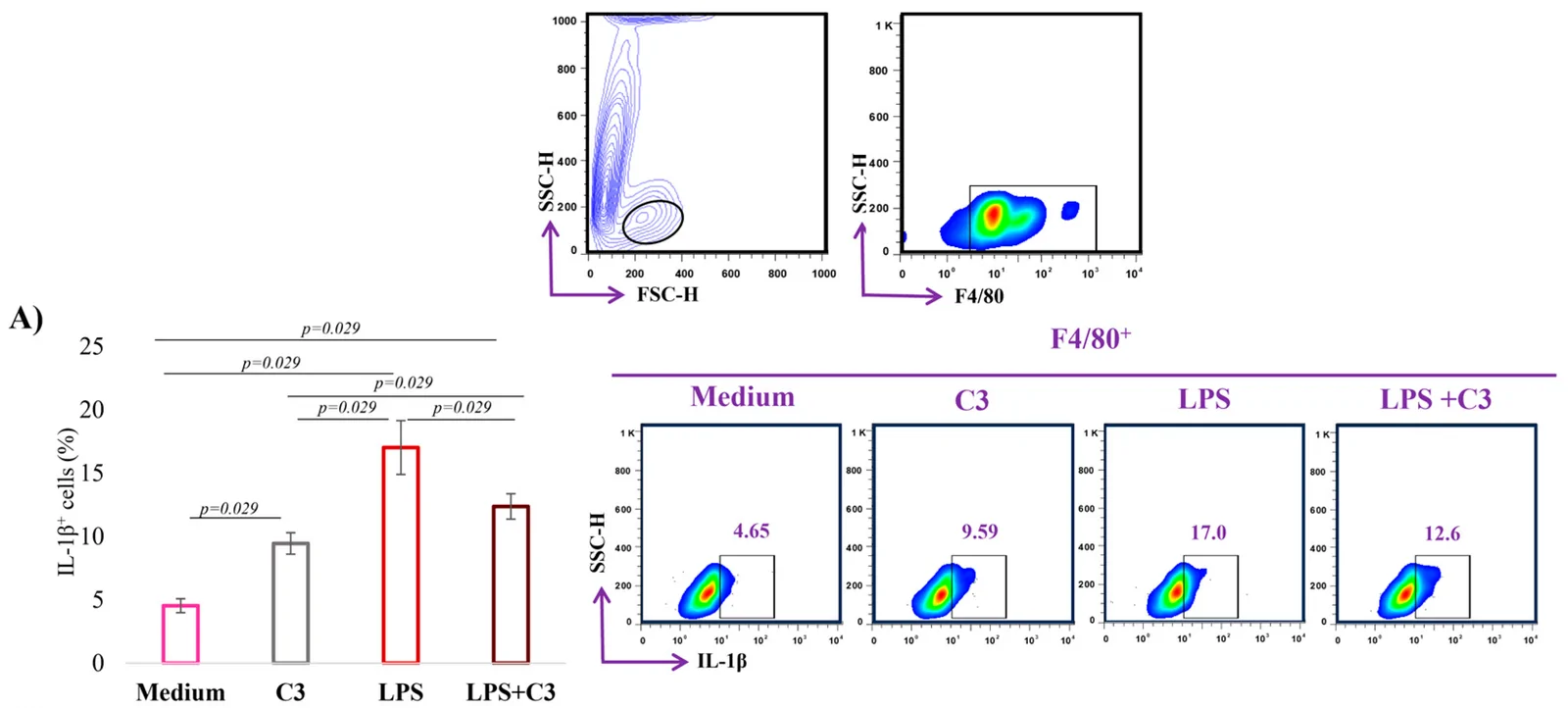

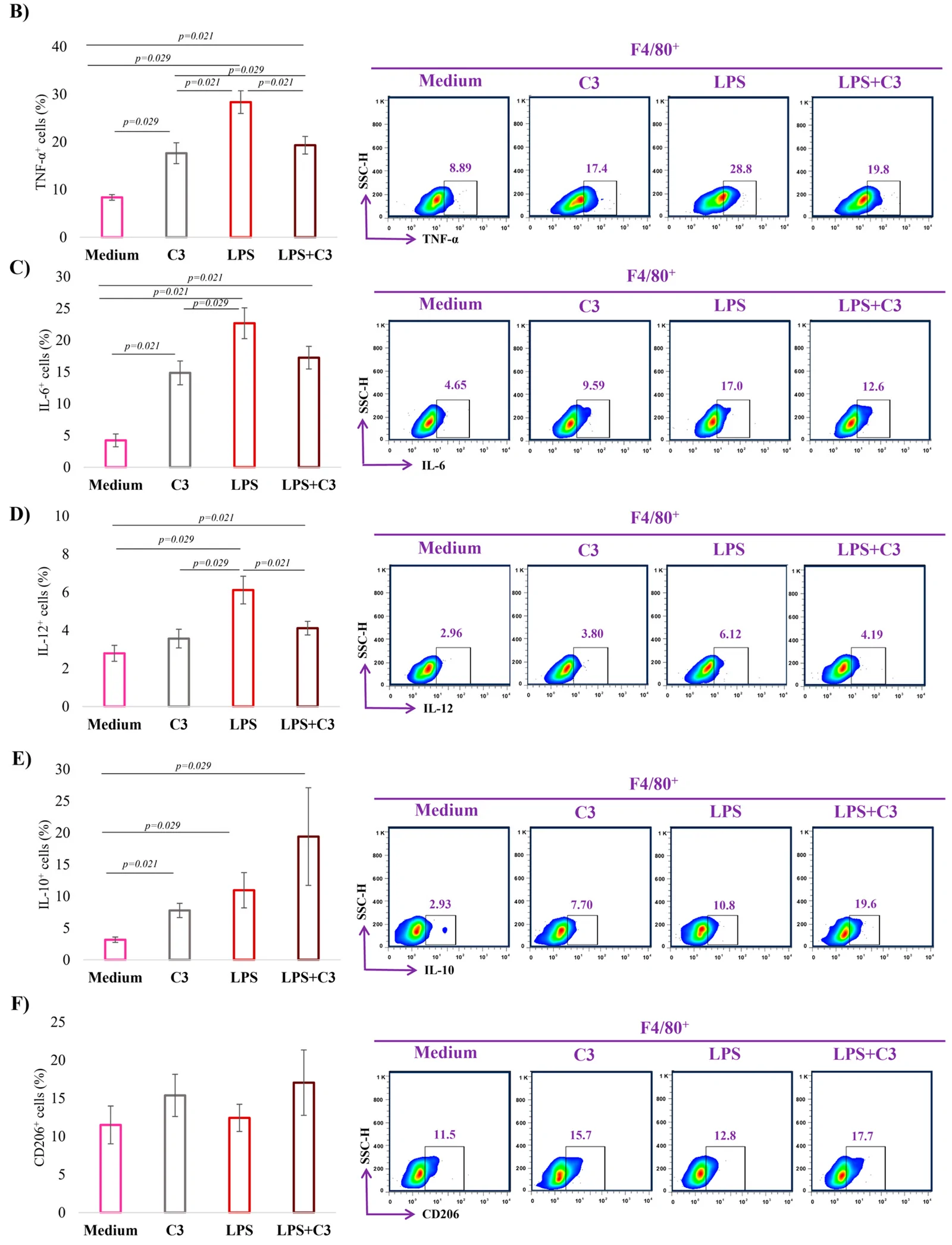
Flow cytometric evaluation of immune markers and cytokines in F4/80 cells after LPS stimulation and/or **C3** complex treatment. Graphs represent the percentage of IL-1β^+^ (**A**), TNF-α^+^ (**B**), IL-6^+^ (**C**), IL-12^+^ (**D**), IL-10^+^ (**E**), and CD206^+^ (**F**) F4/80^+^ BALB/c-derived splenocytes cultivated in the presence of medium, LPS, **C3** complex, or LPS and **C3** complex. Data are presented as mean ± SEM of five mice per group and are representative of three independent experiments. Statistical significance was determined by Kruskal–Wallis test followed by Mann–Whitney U test.

**Figure 7:**
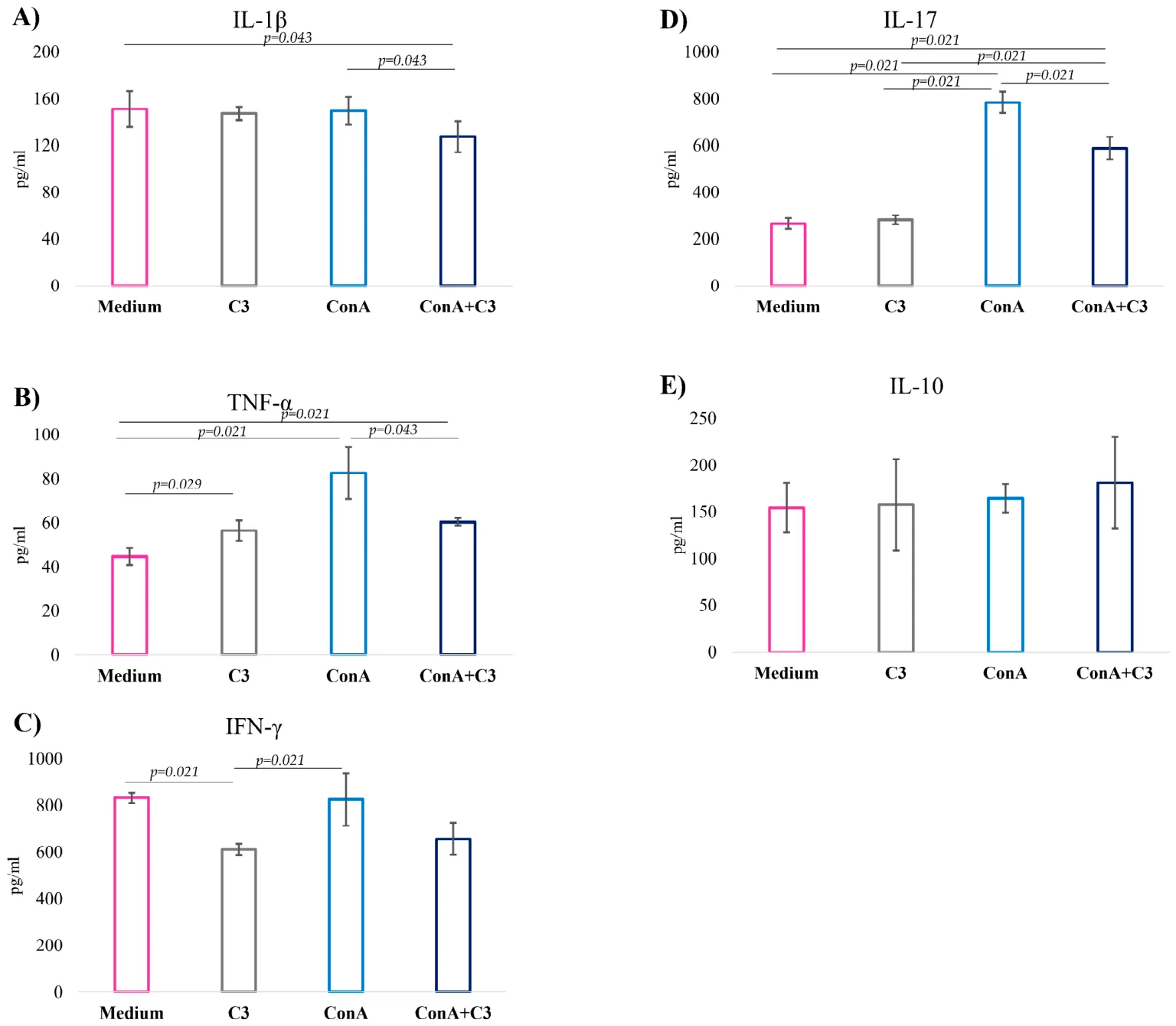
The concentration of IL-1β (**A**), TNF-α (**B**), IFN-γ (**C**), IL-17 (**D**), and IL-10 (**E**) measured in the supernatant of splenocytes isolated from BALB/c mice after cultivation with medium, ConA, **C3** complex, or cocultivation with ConA and **C3** complex. ELISA assay was used to determine cytokine concentrations. Data are presented as mean ± SEM of five mice per group and are representative of three independent experiments. Statistical significance was determined by Kruskal–Wallis test followed by Mann–Whitney U test.

**Figure 8:**
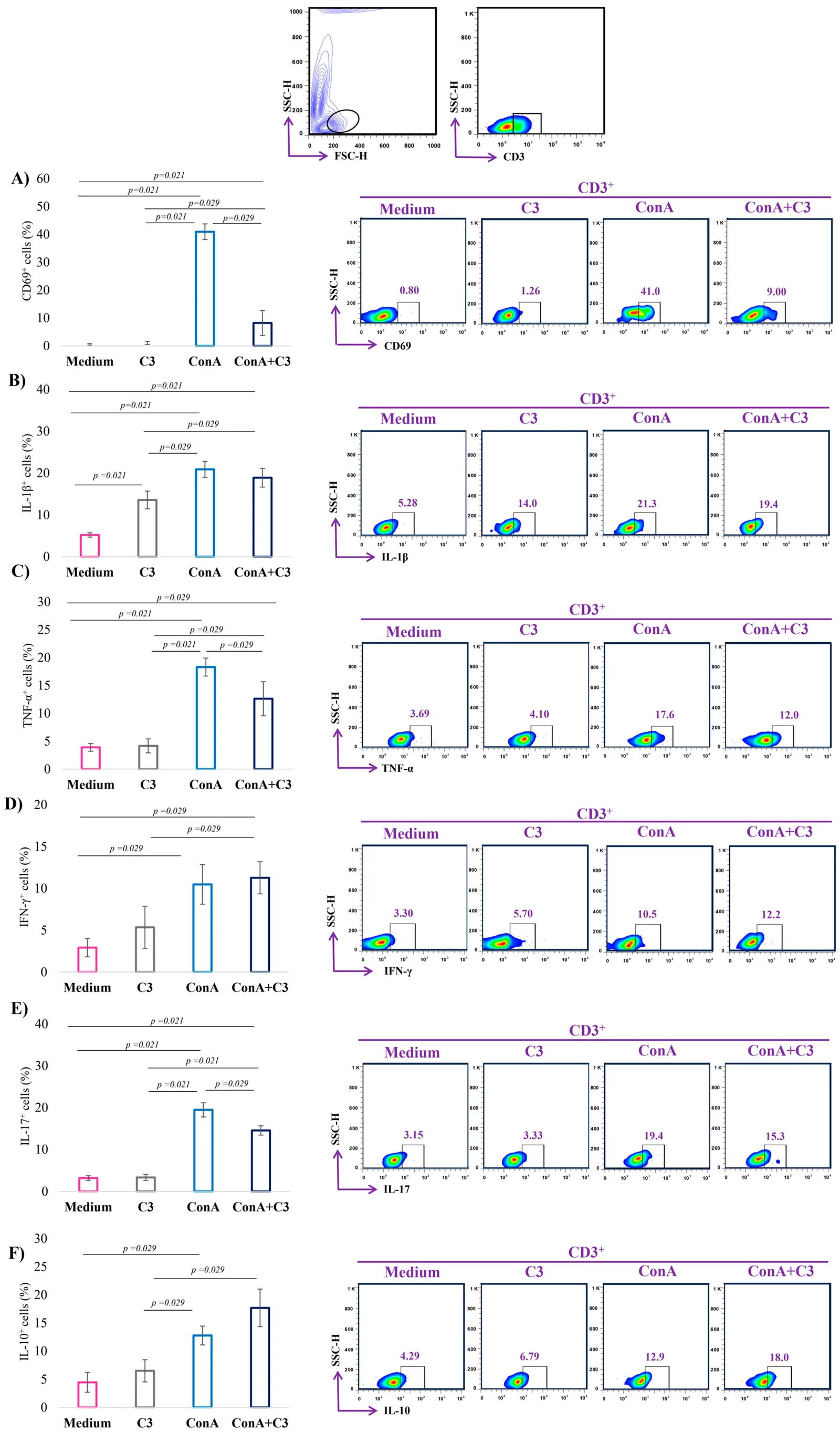
Phenotypic activation and cytokine profile of CD3^+^ T-lymphocytes. Graphs represent the percentage of CD69^+^ (**A**), IL-1β^+^ (**B**), TNF-α^+^ (**C**), IFN-γ^+^ (**D**), IL-17^+^ (**E**), and IL-10^+^ (**F**) CD3^+^ BALB/c-derived splenocytes cultivated in the presence of medium, ConA, **C3** complex, or ConA and **C3** complex. Data are presented as mean ± SEM of five mice per group and are representative of three independent experiments. Statistical significance was determined by Kruskal–Wallis test followed by Mann–Whitney U test.

**Table 1:** Antimicrobial activity of investigated ligands and silver(I) complexes.

Substance/ Microorganisms		*B. subtilis* ATCC 6633	*B. subtilis*	*S. aureus* ATCC 25923	*S. aureus*	*E. coli* ATCC 25922	*E. coli*	*P. mirabilis* ATCC 12453	*P. aeruginosa* ATCC 27853	*S. enterica*	*C. albicans* ATCC 10231
**L1**	MIC	>500	>500	>500	>500	500	500	500	>500	500	>500
	MMC	>500	>500	>500	>500	500	>500	500	>500	>500	>500
**L2**	MIC	>500	>500	>500	>500	250	500	500	>500	500	>500
	MMC	>500	>500	>500	>500	500	>500	500	>500	>500	>500
**L3**	MIC	>500	>500	>500	>500	500	>500	500	500	500	>500
	MMC	>500	>500	>500	>500	500	>500	>500	>500	>500	>500
**L4**	MIC	>500	500	500	>500	500	>500	>500	500	>500	500
	MMC	>500	>500	>500	>500	500	>500	>500	>500	>500	>500
**L5**	MIC	>500	>500	500	>500	250	>500	>500	>500	>500	>500
	MMC	>500	>500	>500	>500	500	>500	>500	>500	>500	>500
**C1**	MIC	62.5	62.5	15.6	62.5	15.6	15.6	15.6	3.9	31.25	62.5
	MMC	62.5	62.5	31.25	62.5	15.6	62.5	62.5	15.6	31.25	250
**C2**	MIC	31.25	31.25	31.25	62.5	7.8	31.25	15.6	3.9	31.25	62.5
	MMC	62.5	62.5	250	62.5	15.6	62.5	62.5	3.9	62.5	250
**C3**	MIC	31.25	125	31.25	250	15.6	125	31.25	3.9	62.5	125
	MMC	62.5	250	250	500	125	250	62.5	15.6	125	250
**C4**	MIC	15.6	62.5	31.25	62.5	15.6	62.5	15.6	3.9	31.25	250
	MMC	62.5	62.5	62.5	62.5	31.25	125	62.5	7.8	62.5	500
**C5**	MIC	15.6	31.25	7.8	62.5	15.6	15.6	7.8	7.8	31.25	31.25
	MMC	62.5	62.5	15.6	62.5	15.6	31.25	31.25	31.25	31.25	62.5
**Tetracycline**	MIC	1.96	0.98	<0.49	<0.06	3.91	1.96	15.6	7.81	0.49	/
	MMC	15.6	1.96	1.96	<0.06	7.81	3.91	31.25	62.5	3.91	/
**Fluconazole**	MIC	/	/	/	/	/	/	/	/	/	31.25
	MMC	/	/	/	/	/	/	/	/	/	62.5

MIC—minimum inhibitory concentration; MMC—minimum microbiocidal concentration. Data are given in µg/mL; /—not tested.

**Table 2:** Activity of silver(I) complexes against preformed bacterial biofilms.

Microorganisms	*P. aeruginosa* ATCC 27853		*P. mirabilis* ATCC 12453		*S. aureus* ATCC 25923	
Silver(I) Complexes/ Antibiotics	BIC_50_	BIC_90_	BIC_50_	BIC_90_	BIC_50_	BIC_90_
**C1**	136	>500	250	>500	128	>500
**C2**	75	>500	216	>500	250	>500
**C3**	98	>500	332	>500	186	>500
**C4**	125	>500	224	>500	310	>500
**C5**	>500	>500	>500	>500	362	>500
**Vancomycin**	734	>1000	785	>1000	63	>1000
**Ceftriaxone**	116	>1000	231	>1000	475	>1000

BIC_50_—concentration required to reduce preformed biofilm biomass by 50%. BIC_90_—concentration required to reduce preformed biofilm biomass by 90%. Data given as µg/mL. BIC_50_ and BIC_90_ values were estimated by nonlinear regression of the mean biofilm inhibition values obtained from three independent experiments, each performed in duplicate. Values reported as >500 µg/mL indicate that 50% or 90% biofilm inhibition was not achieved within the tested concentration range.

**Table 3:** Selected structural and energetic parameters of the optimized Ag(I) complexes **C1**–**C5**.

Compound	Ag-Ag (Å)	Ag-O (Å)	Final Energy (Eh)
**C1**	2.7970	2.1301–2.1408	−2009.2343
**C2**	3.1276	2.1325–2.3540	−2087.8381
**C3**	2.8123	2.1245–2.1390	−2165.0299
**C4**	2.8135	2.1425–2.1470	−2245.0405
**C5**	2.8529	2.1336–2.1811	−2469.5407

**Table 4:** The calculated thermodynamic parameters for the best-docked conformations of silver(I) complexes with LasR.

Complex	ΔG (kcal/mol)	ΔG_vdw+hbond+desolv_ (kcal/mol)	ΔG_elec_ (kcal/mol)	ΔG_Intermol_. _Energy_ (kcal/mol)	ΔG_total_ (kcal/mol)	ΔG_tor_ (kcal/mol)	ΔG_unb_ (kcal/mol)
**C1**	−9.45	−10.56	+0.02	−10.54	−0.40	+1.10	−0.40
**C2**	−10.34	−12.52	−0.02	−12.53	−0.27	+2.20	−0.27
**C3**	−10.20	−12.91	−0.04	−12.94	−0.38	+2.74	−0.38
**C4**	−9.42	−11.73	+0.11	−11.62	−1.45	+2.20	−1.45
**C5**	−9.22	−10.33	+0.01	−10.32	+0.17	+1.10	+0.17

**Table 5:** Predicted ADMET properties of the investigated ligands.

Compound	Intestinal Absorption	BBB Permeability	P-gp Substrate	Skin Permeability	AMES Toxicity	hERG I/II Inhibition
**L1**	93.27%	0.146	No	−2.586	No	No/No
**L2**	92.65%	0.094	No	−2.575	No	No/No
**L3**	92.50%	0.042	No	−2.611	No	No/No
**L4**	92.28%	0.004	Yes	−2.605	No	No/No
**L5**	92.24%	0.081	No	−2.542	No	No/No

## Data Availability

The original contributions presented in this study are included in the article. Further inquiries can be directed to the corresponding authors.
